# Quorum-quenching limits quorum-sensing exploitation by signal-negative invaders

**DOI:** 10.1038/srep40126

**Published:** 2017-01-05

**Authors:** Mélanie Tannières, Julien Lang, Claudie Barnier, Jacqui A. Shykoff, Denis Faure

**Affiliations:** 1 Institute for Integrative Biology of the Cell (I2BC), CNRS CEA Univ. Paris-Sud, Université Paris-Saclay, Avenue de la Terrasse, Gif-sur-Yvette 91198, France; 2 Ecologie Systématique Evolution, CNRS, Univ. Paris-Sud, AgroParisTech, Université Paris-Saclay, 91400 Orsay, France

## Abstract

Some bacteria produce and perceive quorum-sensing (QS) signals that coordinate several behaviours, including the costly processes that are exoenzyme production and plasmid transfer. In the case of plasmid transfer, the emergence of QS signal-altered invaders and their policing are poorly documented. In *Agrobacterium tumefaciens*, the virulence Ti-plasmid encodes both synthesis and sensing of QS-signals, which promote its transfer from a donor to a recipient cell. Here, we reported that QS-altered *A. tumefaciens* mutants arose during experimental evolution. All showed improved growth compared to their ancestor. Genome sequencing revealed that, though some had lost the Ti-plasmid, most were defective for QS-signal synthesis and Ti-plasmid conjugation (*traR* mutations) and one exhibited a QS-signal exploitation behaviour, using signal produced by other cells to enhance its own Ti-plasmid transfer. We explored mechanisms that can limit this QS-hijacking. We showed that the *A. tumefaciens* capacity to inactivate QS-signals by expressing QS-degrading enzyme could attenuate dissemination of the QS signal-negative Ti-plasmids. This work shows that enzymatic QS-disruption whether encoded by the QS-producing Ti-plasmid itself, by a companion plasmid in the same donor cells, or by one in the recipient cells, in all cases can serve as a mechanism for controlling QS exploitation by QS signal-negative mutants.

Quorum-sensing (QS) is a widespread regulatory system in which threshold concentration of a diffusible chemical, called QS-signal, regulates gene expression^1^,^2^. Numerous bacterial species use the QS-pathway to coordinate and synchronize behaviours^3^,^4^. QS-regulated functions are diverse, including biofilm construction, synthesis of antibiotics and virulence factors, and plasmid transfer, the last leading to a genetic exchange between donor and recipient bacterial cells^5^. As a cooperative system, QS is exposed to two kinds of threats. The first is the emergence of individuals that would be blind to QS signals but still would benefit from the QS-regulated functions of others, and in this case we would speak of QS-cheating. The second one is the emergence of QS signal-negative or -attenuated individuals that would use the QS-signals emitted by others to activate, to their own benefit, their own QS-regulated functions, and in this case we would speak of QS-hijacking. Experimental reports and mathematical models document the appearance of these QS-cheating and QS-hijacking subpopulations, especially highlighting their ecological implications^6^,^7^,^8^,^9^,^10^. In parallel, strategies by which QS signal-emitting populations can combat QS-cheating and QS-hijacking are starting to be unveiled^6^,^11^. The involvement of QS-signal degrading enzymes in policing QS-cheating and QS-hijacking is unknown^12^,^13^.

*Agrobacterium tumefaciens* cells can carry a tumour-inducing (Ti) plasmid that codes for virulence. *A. tumefaciens* cause crown gall disease in their host plants by transferring the T-DNA of Ti-plasmid to plant cells, thereby inducing a plant tumour. Expression of the Ti-plasmid encoded virulence functions imposes a strong fitness cost to *A. tumefaciens*^14^,^15^,^16^. It has previously been observed that non-pathogenic *A. tumefaciens* mutants, with impaired transfer of the T-DNA to plant cells and tumour formation, arise in plant tumours and *in vitro* when virulence functions were expressed^14^,^15^,^17^,^18^. The same reports revealed that the responsible mutations may occur in plasmid or chromosomal loci.

The infected tissues produce several plant tumour-specific compounds (called opines) that are consumed by the *A. tumefaciens* population harbouring the Ti-plasmid, hence providing an ideal habitat for the pathogenic agrobacteria^19^. The same Ti-plasmid also codes for QS-signalling, which controls the dissemination of the Ti-plasmid by bacterial conjugation between a virulent Ti-plasmid donor and an avirulent recipient individual, which thereby becomes virulent through Ti-plasmid acquisition^20^,^21^. Ti-plasmid conjugation occurs in the induced plant-tumour tissues, as some opines are required for increasing the transcription of Ti-plasmid genes responsible for the production (*traI*) and sensing (*traR*) of the QS-signal^21^,^22^. Virulent Ti-plasmids thus modify the host-plant habitat of *A. tumefaciens* cells by inducing opine production in infected plant cells and favour their own spread through a QS-controlled horizontal transfer.

In *A. tumefaciens* (also referred as *A. fabrum*) strain C58, two lactonase enzymes are able to cleave QS-signal^23^,^24^. In the plant pathogen *A. tumefaciens* and the animal pathogen *P. aeruginosa* QS-signal degrading enzymes are thought to contribute to recycling the QS-signal and/or preventing its accumulation under nutrient limitation^25^,^26^,^27^,^28^. However, in *A. tumefaciens*, these enzymes also weakly reduce Ti-plasmid transfer during bacterial conjugation^29^,^30^,^31^.

Here we address an as yet unexplored aspect of *A. tumefaciens* QS-signalling, by investigating the emergence of QS-altered mutants affected in virulence and Ti-plasmid transfer. In addition, we explored mechanisms for the control of QS-exploitation by signal-negative invaders, and demonstrated that QS-degrading enzymes can play this original role. Finally, we discuss an integrative model for interpreting the Ti-plasmid dynamics in the *A. tumefaciens* QS-emitting populations in the course of the plant host infection. This work provides a new insight in virulence plasmid dynamics under QS-control in bacterial pathogens.

## Results

### Emergence of individuals with altered QS-conjugation of the Ti-plasmid

**Figure 1:**
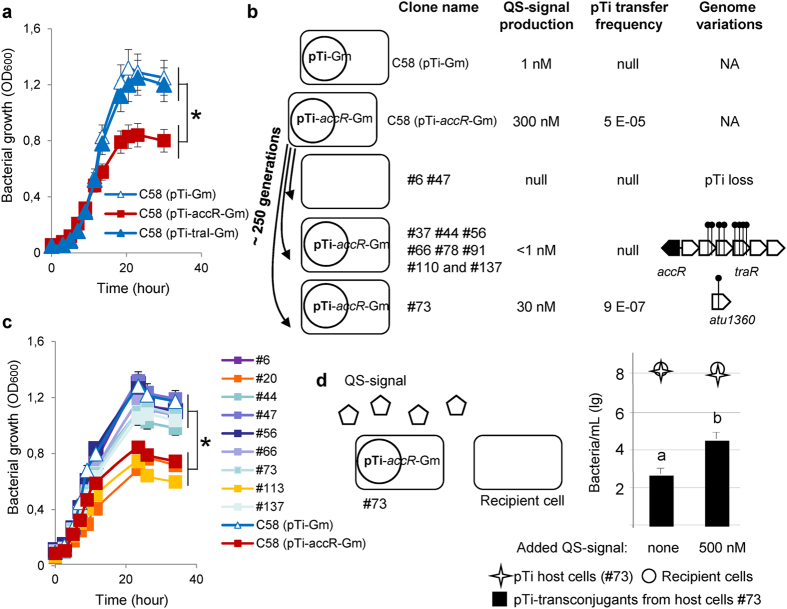
Emergence of QS-mutants. (**a**) growth of the *A. tumefaciens* C58 derivatives carrying the pTi-Gm (no conjugation and wild type low level QS-signal), pTi-*traI*::Gm (no conjugation and defective for QS-signal) and pTi-*accR*::Gm (constitutive AccR-regulon including conjugation and high level QS-signal). The constitutively conjugating strain had significantly lower carrying capacity than the other two strains that did not differ (F_(2,94)_ = 22.36, p < 0.0001, for difference in overall optical density, indicated by an asterisk). The three experimental blocks did not differ significantly F_(2,94)_ = 2.74, p = 0.07. The r^2^ for the model = 0.88. **(a**) main characteristics of the analysed QS-altered mutants (#6, #37, #44, #47, #56, #66, #73, #78, #91, #110 and #137) deriving from the *A. tumefaciens* C58 (pTi-*accR*::Gm) ancestor, and the *A. tumefaciens* C58 (pTi-Gm). In this assay, pTi conjugation was performed in the absence of agrocinopine. NA, non-applicable. (**c**) growth of the *A. tumefaciens* C58 clones carrying the pTi-Gm and pTi-*accR*::Gm, of the evolved QS-altered mutants (#6, #37, #44, #47, #56, #66, #73, #78, #91, #110 and #137) and of two evolved clones (#20, #113) that retained ancestral QS-production. Evolved QS-altered mutants had significantly (indicated by an asterisk) more rapid growth than evolved (clones #20, #113) or ancestral (pTi-*accR*::Gm) clones with high QS-signal production; analysis performed on square-root transformed optical density, respective slopes ± SE for increase in optical density over time 0.0376 ± 0.0007 and 0.0288± 0.0013; interaction time x bacterial genotype F_(1,161)_ = 62.01, p < 0.0001. The two experimental blocks did not differ significantly F_(1,161)_ = 0.27, p = 0.60. The r^2^ for the model = 0.97. (**d**) Ti-plasmid conjugation of the clone #73 in the absence and presence of additional QS-signal (500 nM). Letters indicate statistically different levels of Ti-plasmid transconjugants (Kruskal-Wallis, p < 0.05, n = 3).

To explore the emergence of spontaneous QS-altered mutants, we used the *accR* genetic background (pTi-*accR*::Gm plasmid) of *A. tumefaciens* C58 in which the AccR regulon is constitutively expressed. AccR regulon encompasses QS-regulated genes such as those coding for Ti-plasmid transfer and agrocinopine assimilation and QS-independent genes such as *rctB* and *nocR* genes coding for regulators that enhance horizontal transfer of the second *A. tumefaciens* At-plasmid and opine nopaline assimilation^32^. *A. tumefaciens* cells that harboured the pTi-*accR*::Gm plasmid reached a far lower carrying capacity than cells with either the pTi-Gm (control plasmid) or the pTi-*traI*::Gm plasmid (deficient in QS-signal production), these latter two not differing in growth characteristics (Fig. 1a). These results indicated that an active AccR regulon (that includes QS-regulated Ti plasmid conjugation) negatively affected the growth of the cells.

Because of the fitness cost, we expected QS-altered mutants to emerge under experimental evolution of *A. tumefaciens* carrying the pTi-*accR*::Gm plasmid. Using *A. tumefaciens* carrying pTi-*accR*::Gm plasmid as an ancestor, we propagated 48 independent lineages in liquid cultures, half of them being supplemented with gentamicin to force maintenance of the pTi-*accR*::Gm plasmid. Indeed, after 250 generations, 28 of the 48 analysed lineages included clones presenting loss or reduction of QS-signal production compared to the ancestor. In the 24 lineages evolving in the presence of gentamicin, we found three lineages for which all three tested clones had reduced QS-signal production and nine others with at least one of the three clones showing decreased QS-signal production. In the 24 lineages evolving in the absence of gentamicin, these values were three lineages and 13 lineages respectively. In the evolved clones and lineages in which QS-signal production was not altered, it is of course possible that QS-independent improvement of fitness could arise.

A deeper functional and genome-wide characterization of eleven clones (from eleven independent lineages), ten presenting no QS-signal production (#6, #37, #44, #47, #56, #66, #78, #91, #110 and #137) and one (#73) presenting a tenfold reduction (30 nM instead of 300 nM in the ancestral culture) was then undertaken. All of these clones had impaired ability to conjugate the Ti-plasmid (Fig. 1b). In addition, we compared growth characteristics of ancestral (*A. tumefaciens* harbouring pTi-*accR*::Gm) and evolved clones (#20 and #113) that retained high constitutive QS-signal production with that of clones with low or absent QS-signal production (seven of the eleven above-mentioned evolved clones). In clones with low or null QS-signal production, growth yield was higher than in those with constitutive QS-signal production (Fig. 1c). In these eleven clones, no mutations (Table S2) in QS-independent genes of the AccR-regulon were identified, showing that fitness gain was strictly associated with QS-alteration *per se*. Noticeably, clones #6 and #47, which were recovered from two independent cultures without antibiotic selection, had lost their virulence on plant hosts as well as their ability to assimilate the opine nopaline (Table S2), two traits that are encoded by the Ti-plasmid. Consistently genome sequencing showed that these 2 clones had lost their Ti-plasmid (Fig. 1b and Table S2). This finding revealed that the spontaneous loss of Ti-plasmid constitutes a simple way for the bacterial host to gain fitness in the absence of selection maintaining the Ti-plasmid.

Genome sequencing also showed that all the other derivatives (#37, #44, #56, #66, #78, #91, #110 and #137) except clone #73 exhibited mutations in the *traR*-containing operon of the Ti-plasmid (Fig. 1b and Table S2). The *traR* gene, which codes the QS-signal sensor, is essential for QS-signal synthesis and QS-controlled conjugation. Therefore, it appears that the *traR*-containing operon is a privileged target for diminishing the costs associated with the QS regulon, and hence for improving the fitness of the bacterial hosts in a competitive arena. This phenomenon was found whether the Ti-plasmid was maintained by selection using gentamicin (#78, #91, #110, #137) or not (#37, #44, #56, #66). Therefore, alteration of the TraR master regulator constitutes a second way for the bacterial host to gain fitness.

Using the sequenced eleven derivatives as donor cells, we also tested their Ti-plasmid conjugation ability by adding pure QS-signal (500 nM) to the culture medium. Only clone #73 increased conjugation with added QS-signal (Fig. 1d). The ten other clones were, as expected, insensitive to the extra QS-signal, having lost their Ti-plasmid or a functional *traR* operon. In the #73 clone, genome sequencing revealed a unique non-synonymous change in the chromosomal gene *atu1360* coding for a conserved putative membrane protein (Fig. 1b and Table S2). The function of this protein is not known. Mutant #73 exemplified a third evolutionary outcome: the selection of bacteria that reduce costly expression of QS-regulon but that can use the QS-signals emitted by others for conjugation and propagation of their Ti-plasmid. This may be considered as a type of QS signal-hijacking behaviour.

### QS-signal level and host genetic background modulate QS exploitation by a QS signal-negative mutant.

The above experimental evolution data established that QS-altered variants could emerged from *A. tumefaciens* cells when QS is activated and that genetic background of the host cell could influence QS exploitation in the QS-altered variants. In the next part of the work, we experimentally evaluated how the QS-signal emitting cells could limit QS exploitation by a QS signal-negative mutant through the expression of QS-signal degrading lactonase. Importantly, we tested this policing hypothesis when the QS signal-negative Ti-plasmid was hosted by cells that repress or not QS exploitation.

For these purposes, we used an already constructed Km-marked QS signal-negative plasmid^33^ (named pTiCh) which produced no QS-signal (non-polar *traI* mutation), but could still transfer effectively (wild-type *traR*) into plasmid-free recipient cells in the presence of exogenous QS-signal 3-oxo-octanoylhomoserine lactone but in the absence of agrocinopine (*accR* mutation). The plasmid pTiCh was introduced into the *A. tumefaciens* C58 derivative NTL4 in which the wild-type Ti-plasmid was removed. The plasmid pTiCh was also introduced into the NTL4-TraM derivative which was impaired in the QS-signal perception because of the constitutive expression of the anti-TraR protein TraM.

Then, conjugation assays were performed for comparing transfer properties of the QS signal-negative pTiCh hosted by NTL4 and NTL4-TraM cells in the presence of different concentrations of QS-signal (Figure S1). Horizontal transfer of plasmid pTiCh occurred depending on the concentration of QS-signal, both from donor cells that had an efficient capacity to perceive QS-signals (strain NTL4) and, at a lower level, from donor cells with impaired QS-signal perception (strain NTL4-TraM). However, at the end of the conjugation experiment we found similar concentrations (10^9^ bacteria/mL) of the two introduced cell types, the QS signal-negative and the recipient cells, with only a small number of pTiCh-transconjugants (Figure S1), indicating that neither donors nor recipients were limiting. This conjugation experiment delineated levels of the exogenous QS-signal necessary for conjugation of pTiCh, and showed that different host cell genotypes (NTL4 and NTL4-TraM) differ in QS-signal perception thereby influencing pTiCh transfer.

In the next experiment, we added no synthetic QS-signal to the culture medium. Instead we introduced *A. tumefaciens* C58 derivatives that produced either none, a low (1 nM) or a high level (hundreds of nM) of the QS-signal 3-oxo-octanoylhomoserine lactone because they harboured the plasmid pTi-*traI*::Gm, defective for QS production, pTi-Gm or pTi-*accR*::Gm, respectively (see Table 1). In all the assays, the ratio among the three cell types remained at approximately 1:1:1.

**Figure 2:**
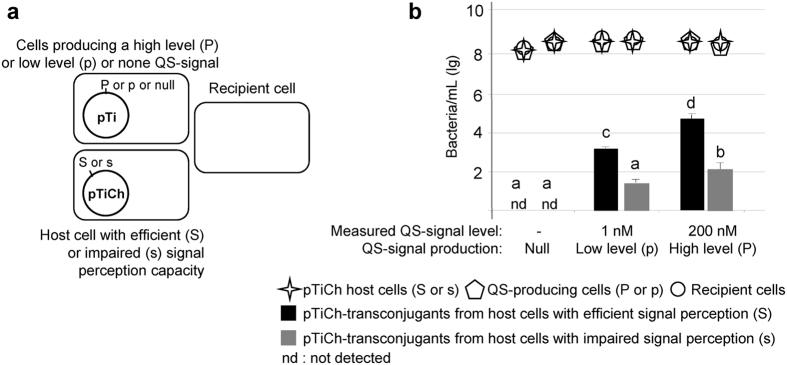
The QS signal-negative plasmid pTiCh can exploit the QS-signals emitted by the QS-signal producing cells. (**a**) experimental setup: cells with efficient (S) or impaired (s) signal perception that host the QS signal-negative plasmid (pTiCh), QS-producing cells which emitted none (pTi-*traI*::Gm), low (pTi-Gm) or high level (pTi-*accR*::Gm) of QS-signals, and recipient cells C58.00 were mixed at 1:1:1 ratio. (**b**) After 24-hour incubation, the pTiCh donor and recipient cells and the pTiCh-transconjugants were counted. Conjugation assays were performed in quadruplicate in two independent blocks. Factorial ANOVA revealed a significant effect of pTiCh host cell (F_(1,64)_ = 387.08, p < 0.0001), signal level (F_(2,64)_ = 281.70, p < 0.0001) and their interaction (F_(2,64)_ = 97.13 p < 0.0001). The two experimental blocks did not differ significantly F_(1,64)_ = 0.32, p = 0.72. The r^2^ for the model = 0.95.

As expected, no pTiCh transconjugants were detected in the absence of QS-signal production but the QS signal-negative plasmid pTiCh transferred into recipient cells when a signal-producer was present (Fig. 2). More plasmid transfer was found at higher signal level (in the presence of *A. tumefaciens* pTi-*accR*::Gm) and for the plasmids in host cells (NLT4) associated with a wild type signal detection capacity. These three-partner assays revealed the capacity of pTiCh to transfer when QS-signal was emitted by other bacteria living in the same environment.

### QS-degradation encoded by QS-producing Ti-plasmid reduces horizontal transfer of the QS signal-negative plasmid

**Figure 3:**
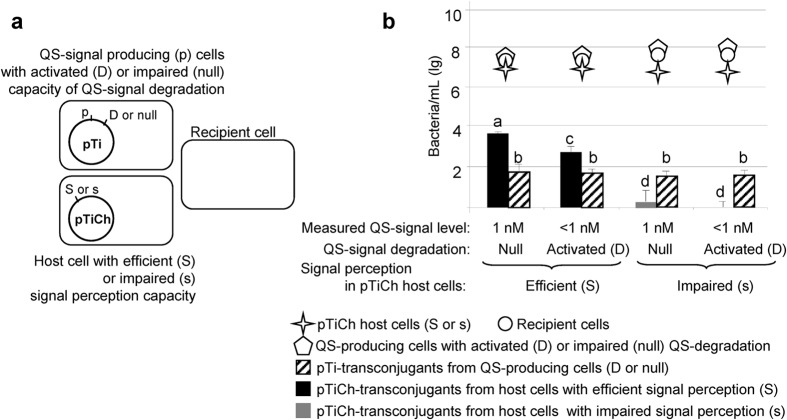
Limitation of QS exploitation by the QS signal-negative plasmid pTiCh when the QS-signal degradation was expressed by the QS-producing plasmid in the pTi donor cells. (**a**) experimental setup: QS-producing cells, which expressed (pTi-Gm) or not (pTi-*aiiB*::Gm) the QS-degrading enzyme AiiB, and recipient cells C58.00 were mixed at 1:1 ratio. Cells with efficient (S) or impaired (s) signal perception and hosting the QS signal-negative plasmid (pTiCh) were added 48 h after to give a final ratio of 1:1:1. All conjugation assays were performed in the presence of synthetic agrocinopine (50 μg/ml) to activate plasmid transfer from QS-producing cells and expression of the lactonase AiiB. (**b**) Twenty-four hours after addition of pTiCh donor cells, the pTiCh-donor, QS-producing and recipient cells and the different transconjugants were counted. Host and recipient cell types per tube remained in an approximately 1:1:1 ratio. Data were collected from 8 replicates in two independent blocks. Producer plasmid transfer did not vary among the different experimental conditions. Factorial ANOVA revealed no significant effects; complete model (F_(4,27)_ = 0.70, p = 0.60). The r2 for the model = 0.09. For QS signal-negative plasmids, factorial ANOVA revealed a significant effect of pTiCh host cell (F_(1,27)_ = 568.51, p < 0.0001), QS-signal degradation (F_(1,27)_ = 29.13, p < 0.0001), and their interaction (F_(1,27)_ = 13.10 p = 0.0012). The two experimental blocks did not differ significantly F_(1,27)_ = 1.88, p = 0.18. The r^2^ for the model = 0.96.

We tested whether QS-degradation enzyme can limit the transfer of the QS signal-negative plasmid that does not produce its own signal (Fig. 3). This experiment was conducted in the presence of pure agrocinopine: this compound induces QS-processes including QS-signal synthesis and plasmid transfer, as well as QS-signal degradation by lactonase AiiB in *A. tumefaciens* C58. We used QS-signal producing cells that hosted one of two types of Ti-plasmids: one expressing the QS-degrading enzyme AiiB (pTi-Gm), the other being defective for this enzyme (pTi-*aiiB*::Gm). Cells that harbour pTi-Gm plasmid encoding the QS-degrading enzyme produce and degrade the QS-signal at the same time and thereby release less QS-signal into the environment as shown in Fig. 3b.

In all the conjugation assays testing for the impact of lactonase, the QS signal-negative pTiCh was added two days after the lactonase and QS-signal producing strains, allowing lactonase-mediated degradation of QS-signal to occur. When pTiCh was harboured by NTL4 host cells with efficient signal detection (Fig. 3b, left columns for “Efficient (S)”), the QS signal-negative pTiCh plasmid transferred ten times less well to recipient cells in the presence of QS-signal degradation. In these same tubes the two QS-signal producer plasmids, with or without QS-signal degradation, transferred at comparable levels under all conditions. This showed that only QS signal-negative plasmids were influenced by QS-degradation. QS-signal that is bound to the QS-sensor TraR is protected from degradation within the producing cell^30^, while most of the free QS-signal will be degraded by lactonase before it exits the cell and becomes available to QS signal-negative cells. Indeed, when the lactonase gene was expressed we found less QS-signal in the culture medium so less was available to cells harbouring a QS signal-negative plasmid.

Globally, the pTiCh transferred more efficiently than did the signal-producing plasmid, when harboured by host cells with efficient signal detection, which was not the case when the pTiCh was harboured by NTL4-TraM host cells with impaired signal detection (Fig. 3b, right columns for “Impaired (s)”), for which we found very few pTiCh transconjugants whether QS-signal was degraded or not.

### When coded on a separate companion plasmid, QS-degradation also reduces transfer of the QS signal-negative plasmid

To test whether QS-signal degradation produced only by a separate plasmid can also impede transfer of a QS signal-negative plasmid under varying levels of signal production, we performed the following experiment. We used plasmids conferring a high (pTi-*accR*::Gm) and low (pTi-Gm) QS-signal production and paired them with a second plasmid placed in the same cell. This second plasmid either expressed (pME*aiiB*) or did not express (pME6010) QS-signal degradation. These four QS-emitting cell types (resulting from a combination of high and low levels of QS-production with or without QS-degradation) were associated with the two pTiCh host cells (Figure S2A). No agrocinopine was added to the culture medium so that QS-degradation activity was not expressed by the producer plasmid, but only by the companion plasmid pME*aiiB* (Table 1).

In all the assays, ratios among these cell types remained at about 1:1:1 and we measured transconjugants that had received the pTiCh. Production of signal-degrading lactonase AiiB on a companion plasmid within the signal-producing cell reduced the amount of QS-signal under both low and high levels of QS-signal production. However, this reduced the number of pTiCh-transconjugants when QS-signal production was low (Figure S2B) but not when QS-signal production was high, conditions in which plasmid transfer was globally higher (Figure S2C). It should be noted that when signal production was high, measured levels of QS-signal, both in presence (60 nM) and absence (400 nM) of degradation, were well above the saturating threshold of 4 nM we found for activation of pTiCh transfer (Fig. 1). This explains that our results for pTiCh transfer were independent of the expression of the QS-signal degradation.

Finally, we posed exactly the same question, with the same experimental set-up, differing only in the placement of the QS-degradation enzyme-coding plasmid. In a final series of experiments we placed this QS-degradation enzyme-coding plasmid pME*aiiB* in the recipient cells (Figure S3A) and found, globally, the same response. QS-degradation enzyme production reduced the number of pTiCh-transconjugants when QS-signal production was low (Figure S3B) but not when it was high (Figure S3C).

## Discussion

This study shows that the QS regulon is costly for *A. tumefaciens*. Cost may arise from signal production and from synthesis, assembly and functioning of the dedicated type-IV secretion system for plasmid transfer^34^. The metabolic costs associated with these processes create conditions that are favourable for the selection of QS-impaired mutants. Among the characterized mutants, we observed, most commonly, the emergence of QS-defective mutants (*traR* mutations) that neither synthesised nor perceived QS-signal and therefore did not transfer Ti-plasmid, but also loss of the Ti-plasmid and emergence of a QS-hijacking mutant with strongly reduced production of QS-signal but maintaining the ability to transfer the Ti-plasmid under the influence of QS-signals emitted by neighbouring cells. The master regulator TraR thus appears the preferential target for reducing the cost associated with the QS-activity, but one could not exclude that mutations in other loci, such as *traI* coding for QS-signal production, may occur. In *Sinorhizobium(Ensifer) meliloti* and *Pseudomonas aeruginosa*, mutations in the genes coding the master QS-regulators ExpR and LasR are also preferentially selected under costly QS-conditions^10^,^35^,^36^.

**Figure 4:**
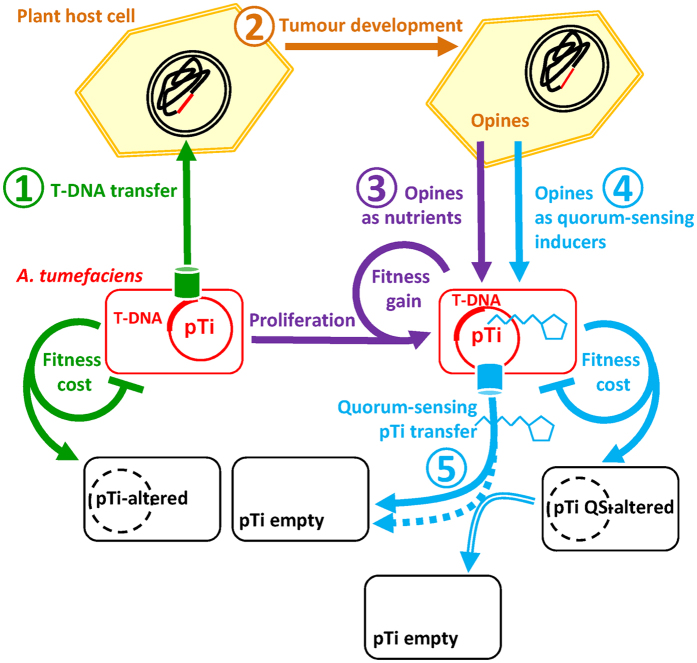
A conceptual model of Ti-plasmid dynamics in the *A. tumefaciens*-induced plant tumour. The model synthesises information from the cited literature and this work to explain the complex dynamics of Ti-plasmids. In step 1, *A. tumefaciens* pathogens transfer T-DNA into a host plant cell. This costly process may select avirulent *A. tumefaciens* individuals, altered for T-DNA transfer, because of Ti-plasmid loss or plasmid and chromosomal mutations. In step 2, T-DNA causes tumour development with production of opines that are consumed by *A. tumefaciens* cells harbouring a Ti-plasmid, leading to their proliferation, in step 3. In step 4, opines induce costly QS-signalling and Ti-plasmid conjugation, which may select QS-altered *A. tumefaciens* individuals impaired in QS-signal production and Ti-plasmid conjugation, leading to the emergence of potential recipient cells lacking a plasmid or harbouring QS signal-negative plasmid. In step 5, QS-signal induces conjugation, spreading Ti-plasmids to cells lacking them. In this step QS-exploiting cells can spread their QS-altered Ti-plasmid by using extrinsic QS-signal. As we show, QS-degrading lactonases reduce available QS-signal that could potentially be used by QS-altered invaders, thereby limiting their spread to the profit of legitimate QS-cooperators.

When infecting plants, another process is costly for *A. tumefaciens*: the transfer of the T-DNA from the Ti-plasmid to the plant cells. The costly activation of this process also contributes to the selection of lower virulence *in vitro* and *in planta* via either Ti-plasmid loss or rearrangements in the Ti and At plasmids or the chromosome^14^,^15^,^16^,^17^,^18^. Altogether, these data suggest that two costly processes, T-DNA transfer and QS regulon occur when *A. tumefaciens* infects and colonizes the plant host. Selection should favour bacterial mutants that minimise these costs, for example, by losing or modifying integrity and conjugation capacity of the Ti-plasmid. On the other hand, the two processes contribute to maintain the Ti-plasmid in the infective population. Firstly, the bacteria harbouring a functional Ti-plasmid have a growth advantage in the opine-rich environment of the plant tumour, because they can consume these opines^19^. Secondly, some opines such as agrocinopines activate QS-activity and Ti-plasmid conjugation, which will lead to Ti-plasmid reintroduction into cells that have lost their Ti-plasmids. This should generate a complex dynamic of repeated cycles of loss and gain, summarized in Fig. 4. Importantly, since cells containing a Ti-plasmid are several orders of magnitude less competent for the acquisition of another one^37^, QS-hijacking limitation is important because the maintenance of QS-producing Ti-plasmids depends largely on their ability to transfer to new host cells that contain no Ti-plasmid.

We explored how the emergence and proliferation of QS-altered individuals in *A. tumefaciens* populations might be limited. A first mechanism may consist of limiting availability of QS-signal to non-producing cells. The main molecular actor of this process would be the regulatory protein TraM, which binds to the QS-master regulator TraR, preventing QS-signal synthesis^33^. Here we propose a complementary mechanism involving the QS-signal degrading lactonase. We show that a QS-signal degrading enzyme can impede the spread of QS signal-negative plasmids that do not encode QS-signal but make use of the QS-signal produced by other individuals to initiate their own transfer to recipient cells. The QS-signal degrading enzyme reduced the spread of QS signal-negative plasmids whether it was produced in *cis* by the QS-signal producing plasmid itself (Fig. 3), in *trans* by a companion plasmid in the same cells (Figure S2) or even in an independent recipient cells (Figure S3), while not impeding the transfer of the Ti-plasmids from QS-signal-producing cells (Fig. 3), consistent with previous observations^28^,^30^. The fact that QS-degrading enzymes affect only the transfer of QS signal-negative Ti-plasmids and not that of QS-producing plasmids can be explained at a mechanistic level. Indeed when QS-signal is bound to the TraR sensor, it is protected from lactonase-mediated degradation^30^. Cells hosting the QS signal-negative plasmid had only access to extracellular QS-signal (unbound to TraR) that diffused from the producing cells and could therefore be degraded by lactonase (Fig. 3). In other QS-bacteria such as the opportunistic pathogen *P. aeruginosa*, QS-degrading enzymes^25^,^26^,^27^ may also contribute to policing QS-cheating or QS-hijacking behaviours.

This work reveals a novel paradigm involving QS-degrading enzymes for limiting QS exploitation by selfish invaders in cooperative bacterial populations. Given the broad distribution of QS-degrading enzymes in bacteria we suspect that this phenomenon might be of special importance to limit crosstalk between different QS system using the same QS signals in complex microbiomes. The QS-exploitation protection we describe could be complementary with the expected frequency-dependent dynamics of QS signal-negative and QS-emitting plasmids among bacterial populations^9^,^38^ and the possibility of horizontal transfer of the QS-genes for converting hijackers into QS-cooperating partners^39^. We consider that conversion by horizontal transfer is unlikely in the case of *A. tumefaciens* because cells containing a Ti-plasmid are far less competent for the acquisition of a novel Ti-plasmid^37^. More generally additional factors may influence plasmid transfer and susceptibility to QS exploitation by signal negative plasmids. We found that different host genotypes differ in how QS signal-negative Ti-plasmids perceive and use the QS-signal for their own transfer. Finally, environmental heterogeneity may also affect the dynamics of QS signal-negative plasmids and cells by exerting variable selection pressures on collective behaviours^40^. In natural environments, animal and plant hosts, as well as several microorganisms in the associated microbiota produce a variety of compounds that may interact with QS-signal and its degradation^41^,^42^,^43^, thereby modifying the conditions of QS-plasmid transfer.

## Material and Methods

### Bacterial strains and plasmids

All bacterial strains used were derivatives of *A. tumefaciens* C58 each carrying modified Ti-plasmids. The four gentamicin-resistant plasmids differed in signal production and degradation characteristics^28^ (Table 1). The control plasmid (pTi-Gm) has wild type production and degradation of QS-signal. The plasmid pTi-*aiiB*::Gm has wild type production of QS-signal but is defective for QS-signal degrading lactonase AiiB. Host cells harbouring these two plasmids (pTi-Gm and pTi-*aiiB*::Gm) conjugate only in the presence of the opine agrocinopine. The pTi-*traI*::Gm is completely defective for QS-signal synthesis and pTi conjugation. The plasmid pTi-*accR*::Gm produces QS-signal and conjugates constitutively, even in the absence of agrocinopine, and thus pays any costs associated with QS under all conditions. This plasmid was used in the experimental evolution lines described below.

The QS signal-negative plasmid (pTiC58*ΔaccRΔtraI-Km* = pTiCh) that harbours a kanamycin resistance was provided by Prof S.K. Farrand^33^. This plasmid produces no QS-signal because of a non-polar deletion in the *traI* gene. However, it is particularly sensitive to QS-signal because of a mutation in *accR* gene that leads to overproduction of the QS-signal sensor TraR. Therefore, in the presence of exogenous QS-signal, this plasmid initiates its transfer even in the absence of agrocinopine^33^. The pTiCh plasmid was introduced by electroporation into an *A. tumefaciens*-C58 derivative (NTL4) from which the wild-type Ti-plasmid had been removed^44^. This same pTiCh plasmid was introduced into a second bacterial host (NTL4-traM), which had ectopic, constitutive expression of the anti-TraR protein TraM, thereby impeding signal sensing by the plasmid pTiCh. This generates two *A. tumefaciens* strains NTL4(pTiCh) and NTL4-traM(pTiCh) which produce no signal and exhibit an efficient and impaired signal perception of the QS-signal, respectively.

The strain C58.00, which contains no plasmid but harbours a chromosomal resistance to rifampicin, was used as a recipient strain throughout. Two other tetracycline-resistant plasmids, the empty vector pME6010 and its derivative pME*aiiB* with constitutive expression of QS-degrading activity were introduced by electroporation in appropriate strains^24^. All the above strains were used in the conjugation experiments that tested invasion ability of the QS signal-negative plasmid (pTiCh).

The antibiotics gentamicin, kanamycin, rifampicin and tetracycline were added to culture media at 25 mg/L, 50 mg/L, 100 mg/L and 10 mg/L respectively.

### Experimental evolution

From the parental *A. tumefaciens* derivative carrying the pTi-*accR*::Gm plasmid, with constitutive QS-signal production and conjugation, 48 parallel cultures were launched in Luria-Bertani modified medium with 5 g/L NaCl with (24 lineages) and without (24 lineages) gentamicin at 25 μg/mL. The gentamicin forces the maintenance of the pTi-*accR*::Gm plasmid. These cultures were subcultured every day in a 20 times dilution. After about 250 generations, estimated from changes in population size, we isolated 3 descendant clones from each culture, i.e. a total of 144 descendant clones, using serial dilutions. Clones numbered #1 to #72 were collected from the 24 lineages without gentamicin and #73 to #144 from the 24 lineages with gentamicin selection. We tested QS-signal production for all these 144 evolved clones against a standard of the pure QS-signal 3-oxo-octanoylhomoserine lactone (Sigma-Aldrich) as previously described using the bio-indicator strain *A. tumefaciens* NT1(pZLR4)^45^,^46^.

### Comparing evolved and parental strains

We conducted a more detailed comparison of eleven clones (from eleven independent lineages), ten presenting no QS-signal production (#6, #37, #44, #47, #56, #66, #78, #91, #110 and #137) and one (#73) presenting a tenfold reduction (30 nM instead of 300 nM in the ancestral culture). Total genomic DNA was extracted and purified with the DNeasy Blood and Tissue kit (Qiagen) according to the manufacturer’s instructions. For each analysed clone, paired-end libraries (2 × 100) were prepared from 5 μg of total genomic DNA using the TruSeq SBS Kit v3 - HS 200-cycles (FC-401-3001, Illumina). Hiseq sequencing was performed at the I2BC platform (Gif-sur-Yvette, France) and the data were analysed through the CASAVA-1.8.2 (demultiplexing), Fastqc 0.10.1 (read quality), and Cutadapt-1.3 (adaptor trimming) pipeline. Sequence reads were mapped onto the annotated reference genome of *A. tumefaciens* C58. Mappings were carried out using the CLC Genomics Workbench v7.5 (CLC bio, Aarhus, Denmark) with threshold values of 90% for read length and 95% for sequence similarity. Genomic variant detection was processed using CLC Genomics Workbench with a minimum coverage of 10 and a variant probability of 98%.

For these same 11 clones we tested their ability to replicate with the opine nopaline as sole nutrient source, their conjugation ability with the recipient strain C58.00 described above, and their virulence against *Arabidospis thaliana*. To test nopaline assimilation ability we grew two replicates of each clone in tubes and measured optical density after 16 hours. Conjugation ability methods are described below in detail. For the virulence test *A. thaliana* (Columbia, Col0) plants were germinated in the greenhouse and then transferred to a controlled environment chamber (22 °C, short day condition, 65% hygrometry). About 3–4 week-old plants were needle-wounded at the base of the stem and infected with the *A. tumefaciens* ancestor C58 (pTi-*accR*::Gm) and its derivatives, using three plants per tested clone. *A. tumefaciens* inoculum was prepared on LBm agar medium and collected with a pipette tip. Tumours were observed 28 days post infection.

### Ti-plasmid transfer assays

We tested whether a QS signal-negative plasmid, pTiCh described above, could invade populations of recipient cells under different conditions of QS-signal production, signal perception and signal degradation.

Cells were cultivated as follows: Cells bearing signal-producing plasmids, pTiCh plasmids, or plasmid-free (recipient for plasmid transfer) cells were cultivated at 28 °C under shaking in LBm medium. After an overnight culture, we diluted all cultures to equalize bacterial cells densities at 1.5 OD_600nm_. For the conjugation assays we inoculated 5 μL of signal-producing and recipient populations into 250 μL of the synthetic AB medium^47^ supplemented with mannitol (2 g/L) and NH_4_Cl (1 g/L) in 96-well plates and incubated them at 24 °C. Five μL of the same OD of strains bearing the plasmid pTiCh were introduced either immediately or two days later for the cases where lactonase-mediated degradation of QS-signal was evaluated. This delay allowed the QS-signal to be degraded by the lactonase. For one conjugation assay that did not include the constitutive pTi-*accR*::Gm we added synthetic agrocinopine-A (50 mg/mL) to activate QS-processes, synthesized as previously described^48^.

Twenty-four hours after mixing the pTiCh-harbouring cells with other bacteria, a serial dilution was plated on selective LBm-agar containing the appropriate antibiotics (gentamicin, kanamycin, rifampicin and tetracycline) and growing colonies were counted. Each experiment consisted of four replicates repeated in one to three independent blocks. The C58.00-recipient cells that acquired a new antibiotic resistance were assumed to have acquired a Ti-plasmid by conjugation. Using PCR-primers (Table S1), we verified the presence of the appropriate plasmid by genotyping a sample of approximately 30 putative transconjugants per experimental condition.

Because the Ti-plasmid has a self-incompatibility system, recipient cells which acquired one Ti-plasmid are less competent by several orders of magnitude for acquiring another one^37^, hence co-transfer of the pTi and pTiCh could not be measured in the experiments.

### Statistical analysis

For estimation of the cost of QS-activity we compared bacterial population size, estimated as optical density, measured from three independent replicates at each of 11 different time points, among a wild-type strain, the QS-deficient strain that harboured the plasmid pTi-*traI*::Gm and the strain with constitutive QS-signal production that harboured the plasmid pTi-*accR*::Gm. An Analysis of Covariance (ANCOVA) allowed us to test for differences among the different strains in their overall mean population size and in their growth rates. We similarly compared bacterial population size, estimated as optical density, measured from two independent replicates at each of nine different time points between evolved QS+ and QS− strain types using nested ANCOVA, with individual strains nested within their strain-type. In QS signal-negative plasmid assays, where each experiment consisted of four replicates repeated in one to three independent blocks, we tested whether plasmid transfer differed with level of QS-signal, QS-signal degradation activity and between the two types of pTiCh-hosting cells using a factorial ANOVA followed by Tukey multiple comparison test or, for simple comparisons, a Kruskal-Wallis non-parametric test. For all tests experimental blocks were included as fixed factors and optical density was square-root transformed to improve the normality of the residuals. Analyses were performed with the statistical package JMP^®^ version 12. In the figures showing conjugation assays we present the means with error bars depicting the standard deviation calculated over all blocks and replicates.

## Additional Information

**How to cite this article**: Tannières, M. *et al*. Quorum-quenching limits quorum-sensing exploitation by signal-negative invaders. *Sci. Rep.* **7**, 40126; doi: 10.1038/srep40126 (2017).

**Publisher's note:** Springer Nature remains neutral with regard to jurisdictional claims in published maps and institutional affiliations.

## Supplementary Material

Supplementary Dataset 1

## Figures and Tables

**Table 1:** Characteristics of the QS-producing and QS-degrading plasmids.

Plasmid	Characteristics	Antibiotic resistance	QS-controlled conjugation	QS-signal production	QS-signal perception	QS-signal degradation	Conjugation assay
pTi-*traI*::Gm	*traI*^-^ *traR*^+^ *aiiB*^+^	Gm	−	−	+	enhanced by agrocinopine	Fig. 2
pTi-Gm	*traI*^+^ *traR*^+^ *aiiB*^+^	Gm	+	+	+	enhanced by agrocinopine	Figs 2, 3 and S2,S3
pTi-*accR*::Gm	*traI*^+^ *traR*^c^ *aiiB*^+^	Gm	+	++	++	enhanced by agrocinopine	Figs 2 and S2, S3
pTi-*aiiB*::Gm	*traI*^+^ *traR*^+^ *aiiB*^-^	Gm	+	+	+	−	Fig. 3
pME6010	Companion plasmid	Tc	−	−	−	−	Figs S2 and S3
pME*aiiB*	Companion plasmid pME6010 expressing the lactonase AiiB	Tc	−	−	−	constitutive	Figs S2 and S3
