# Supplementary material for: Quorum-quenching limits quorum-sensing exploitation by signal-negative invaders
Source: Sci Rep. 2017 Jan 5;7:40126. doi: 10.1038/srep40126 (PMC5215187; doi:10.1038/srep40126)

Quorum-quenching limits quorum-sensing exploitation by signal-negative invaders

Mélanie Tannières[1*], Julien Lang[1*], Claudie Barnier[1], Jacqui A. Shykoff[2] and Denis Faure[1]

1 Institute for Integrative Biology of the Cell (I2BC), CNRS CEA Univ. Paris-Sud, Université Paris-Saclay, Avenue de la Terrasse, Gif-sur-Yvette 91198, France

2 Ecologie Systématique Evolution, CNRS, Univ. Paris-Sud, AgroParisTech, Université Paris-Saclay, 91400 Orsay, France

Supporting information

S1 Table: Primers used in this study

S2 Table: Characteristics of the QS-altered mutants

S1 Figure. Transfer of the QS signal-negative plasmid pTiCh requires QS-signal.

S2 Figure. Limitation of QS exploitation by the QS signal-negative plasmid pTiCh when the QS-signal degradation was expressed by a companion plasmid in the QS-producing cells

S3 Figure. Limitation of QS exploitation by the QS signal-negative plasmid pTiCh when the QS-signal degradation was expressed by a companion plasmid in the recipient cells

Supporting tables

Table S1: Primers used in this study

Plasmid	Gene	Primers
pTi-Gm	*atu6148*	5′-atctatcacctgaactacggctat<br>5′-aaaggtctgtcctgtgccaggtcg
pTi-*accR*::Gm	*accR*	5′-gggcaaaagcatctattcggtcgg<br>5′-gcggctcgaaacttcgatcatggc
pTi-*traI*::Gm	*traI*	5′-agaagtctgacgcatccggcgacc<br>5′-cgtgagatgatttctgttcgcatg
pTi-*aiiB*::Gm	*aiiB*	5′-gtcctgtcgcatcttcacaa<br>5′-atgcggtttgaggtagaggc
pTiCh	*traI*	5′-cgtgagatgatttctgttcgc<br>5′-agaagtctgacgcatccggcgacc

Table S2: Characteristics of the QS-altered mutants

The abilities of each clone to produce the QS-signal 3-oxo-octanoylhomoserine lactone, to assimilate nopaline as sole source of C and N and to induce plant tumors (virulence) are indicated by the signs + and -. The genome variations indicate, in this order, the locations of the mutations (atu gene code), the type of mutation (deletion, conversion, stop codon* or frame shift) and the (putative) functions of the encoded protein when known. In bold are highlighted the mutations affecting the *traR* operon. Annotations are from the Agrobacterscope library https://www.genoscope.cns.fr/agc/microscope/home/

Clones	QS-signal production	Nopaline assimilation	Virulence	Genome variations
C58 (pTi-*accR*::Gm)	300 nM	+	+	ancestor
#6	-	-	-	- *atu2592* : Trp245Arg in RedA hypothetical protein<br>- *atu2780* : Ile285Thr in ProB Glutamate 5-kinase<br>- **pTi loss**
#37	-	+	+	- *atu3687* : Asp650Gly in FecA putative iron transporter<br>- **Atu6136 : Val305 frame shift in Na+/phosphate symporter (upstream of *traR*)**
#47	-	-	-	- *atu1364* : Thr261Ala in ClpA ATP-dependent Clp protease, ATP-binding subunit<br>- **pTi loss**
#56	-	+	+	- *atu1888* : Ser289Pro in two component sensor kinase<br>- *atu3687* : Thr250Ala in putative TonB-dependent receptor (iron transport)<br>- *atu4219* : Ala425Thr in putative AcrA family cation<br>- ***atu6134* : Leu233Pro in transcriptional activator protein TraR**
#78	-	+	+	- *atu4898* : His24Arg in hypothetical protein<br>- **deletion (1571nt) between *atu6135* and *atu6136* (upstream of *traR*)**
#91	-	+	+	- *atu4048* : Glu14Ala in 2-hydroxyacid dehydrogenase<br>- ***atu6136* : Leu365 and Lys366 replacement by Phe and stop codon in Na+/phosphate symporter (upstream of *traR*)**
#110	-	+	+	- *atu4442* : Thr18Ile in putative protease<br>- ***atu6135* : Ala335 frame shift in sucrose phosphorylase (upstream of *traR*)**
#137	-	+	+	**- *atu6134* : Val50Ala in transcriptional activator protein TraR**
#44	-	+	+	- *atu3687* : Asp650Gly in FecA putative iron transporter<br>- ***atu6134* : Met1 deletion in transcriptional activator protein TraR**
#66	-	+	+	- *atu3687* : Leu206Ile in FecA putative iron transporter<br>- ***atu6134* : Arg91* in transcriptional activator protein TraR**
#73	30 nM	+	+	- *atu1360* : Glu117Lys in conserved hypothetical protein Atu1360

Supporting figures

Figure S1. Transfer of the QS signal-negative plasmid pTiCh requires QS-signal.

a, experimental setup: cells with efficient (S) or impaired (s) signal perception that host the QS signal-negative plasmid (pTiCh) and recipient cells (C58.00) were mixed at a 1:1 ratio in the presence of QS-signal 3-oxo-octanoylhomoserine lactone at different concentrations (from 0 to 10 nM).

b, After 24-hour incubation, the pTiCh donor, the recipient cells and pTiCh-transconjugants were counted. Conjugation assays were performed in quadruplicate and the experiment was repeated in two independent blocks. Cells with efficient signal perception transferred more pTiCh plasmids than cells with impaired signal perception, ($F_{(1,36)}$=457.58, $p<0.0001$) and plasmid transfer increased with increasing QS-signal concentrations ($F_{(5,36)}$=321.94, $p<0.0001$), more rapidly and to a higher level for cells with efficient signal perception (interaction; $F_{(5,36)}$=46.05, $p<0.0001$). The r^2 for the model =0.98.

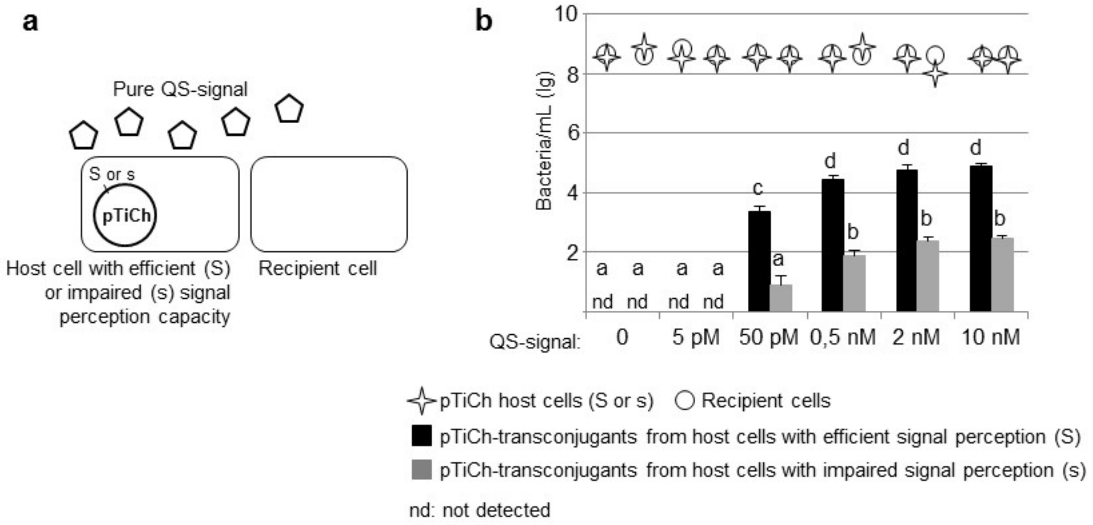

Figure S2. Limitation of QS exploitation by the QS signal-negative plasmid pTiCh when the QS-signal degradation was expressed by a companion plasmid in the QS-producing cells

a, QS-producing cells harbouring the pTi-Gm or pTi-*accR*::Gm, which synthetized a low (panel **b**) or high (panel **c**) level of QS-signals respectively, and recipient cells C58.00 were mixed in a 1:1 ratio. Cells with wild-type (S) or impaired (s) signal perception and hosting the QS signal-negative plasmid (pTiCh) were added after 48 hours to give a final ratio of 1:1:1. The companion plasmids, pME*aiiB* and pME6010, which expressed or not the QS-degrading lactonase AiiB respectively, were hosted by QS-producing cells. After 24-hour incubation of the three cell types together we counted pTiCh-transconjugants.

In **b**, Factorial ANOVA of plasmid transfer under low signal conditions revealed a significant effect of pTiCh host cell ($F_{(1,42)}$=87.70, p<0.0001), and QS-signal degradation ($F_{(1,42)}$=8.50 p=0.0057) but the interaction was not significant ($F_{(1,42)}$=0.02, p=0.88). The three experimental blocks were significantly heterogeneous, $F_{(2,42)}$=25.91, p<0.0001, with the second block showing very little transfer. The r^2 for the model = 0.779. Only transconjugant numbers are shown.

In **c**, under high signal production, factorial ANOVA revealed a significant effect only of pTiCh host cell $F_{(1,42)}$=70.90 p<0.0001, no effect of QS-signal degradation ($F_{(1,42)}$=0.04 p=0.85). The interaction was barely non-significant $F_{(1,42)}$=3.86 p=0.056). Blocks once again were significantly heterogenous ($F_{(2,42)}$=9.03 p=0.0005) r^2=0.69. Only transconjugant numbers are shown.

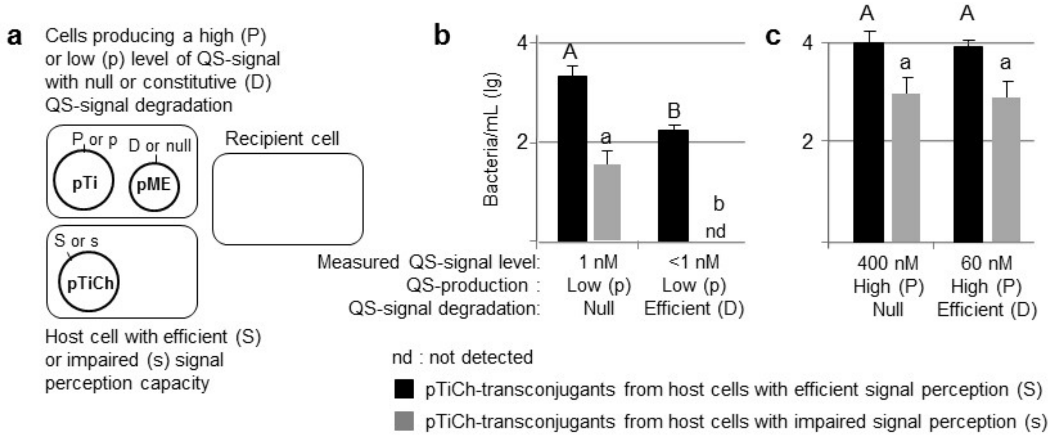

Figure S3. Limitation of QS exploitation by the QS signal-negative plasmid pTiCh when the QS-signal degradation was expressed by a companion plasmid in the recipient cells

a, QS-producing cells harbouring the pTi-Gm or pTi-*accR*::Gm, which synthetized a low (panel **b**) or high level (panel **c**) of QS-signals respectively, and recipient cells C58.00 were mixed in a 1:1 ratio. Cells with wild-type (S) or impaired signal perception (s) hosting the QS signal-negative plasmid (pTiCh) were added 48h after for a final ratio of 1:1:1. The companion plasmids, pME*aiiB* and pME6010, which expressed or not the QS-degrading lactonase AiiB respectively, were hosted by recipient cells. After 24-hour incubation of the three partners we counted pTiCh-transconjugants.

In **b**, factorial ANOVA of plasmid transfer under low signal conditions revealed a significant effect of pTiCh host cell ($F_{(1,27)}$= 461.88, p<0.0001), and QS-signal degradation ($F_{(1,27)}$=12.15 p=0.0017) and the interaction was significant ($F_{(1,27)}$=10.34, p=0.0034). The two experimental blocks did not differ significantly, $F_{(1,27)}$=1.59, p=0.22. The r^2 for the model =0.947. Only transconjugant numbers are shown.

In **c**, under high signal production, factorial ANOVA revealed a significant effect only of pTiCh host cell $F_{(1,12)}$=622.95 p<0.0001, no effect of QS-signal degradation ($F_{(1,12)}$=0.12 p=0.73) or their interaction ($F_{(1,12)}$=0.11 p=0.74) r^2=0.98. Only transconjugant numbers are shown.

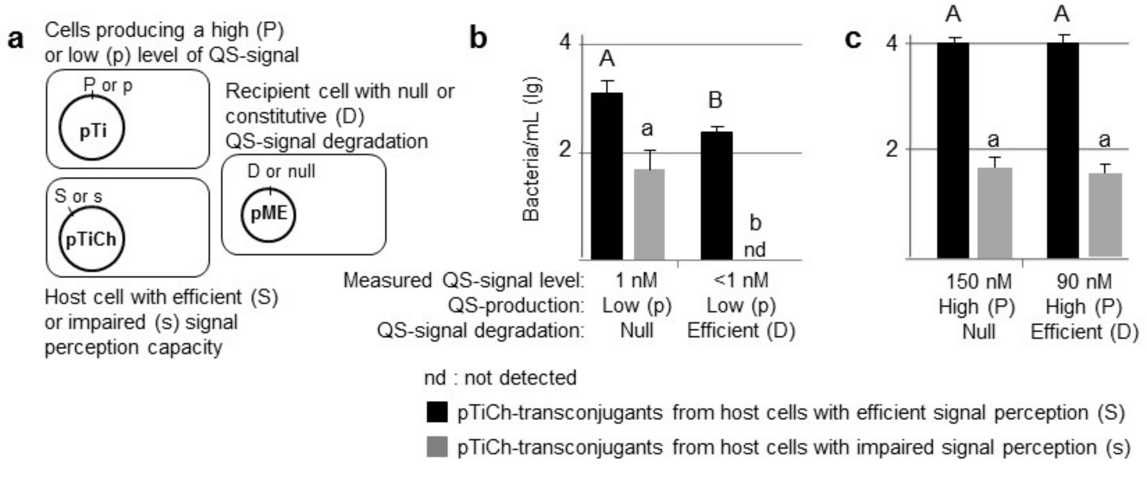

Supplement: Supplementary Dataset 1 [file srep40126-s1.pdf]
